# Efficacy of a group-based brief tobacco intervention among young adults aged 18–20 years in the US Air Force

**DOI:** 10.18332/tid/143282

**Published:** 2021-12-08

**Authors:** Melissa A. Little, Xin-Qun Wang, Margaret C. Fahey, Kara P. Wiseman, Kinsey Pebley, Robert C. Klesges, Gerald W. Talcott

**Affiliations:** 1 School of Medicine, University of Virginia, Charlottesville, United States; 2 University of Virginia Cancer Center, Charlottesville, United States; 3 Department of Psychology, University of Memphis, Memphis, United States; 4 Wilford Hall Ambulatory Surgical Center, 59th Medical Wing, Lackland, United States

**Keywords:** tobacco, young adults, military, cessation, ENDS

## Abstract

**INTRODUCTION:**

Most smokers begin using tobacco before the age of 25 years, making it important to reduce tobacco use during adolescence and early adulthood. Rates of use are historically higher among military personnel. While ‘Tobacco 21’ made it illegal for US retailers to sell tobacco to those aged <21 years, the policy did not address cessation for current youth and young adult tobacco users. Additionally, there is limited research on cessation interventions among young adults under 21 years. The current study evaluated the efficacy of a group-based Brief Tobacco Intervention (BTI) among US Air Force trainees, who are predominantly aged 18–20 years and directly impacted by Tobacco 21 legislation.

**METHODS:**

Participants were 2969 US Air Force Trainees from April 2017 through January 2018 cluster randomized to three conditions: 1) BTI + Airman’s Guide to Remaining Tobacco Free (AG), 2) AG alone, and 3) the National Cancer Institute’s Clearing the Air (CTA) pamphlet. To assess the efficacy of the interventions among people aged 18–20 years, a domain analysis (<21 years, n=2117; and ≥21 years, n=852) of a multinomial logistic regression model was run.

**RESULTS:**

Mono tobacco users aged <21 years at baseline who received the BTI+AG had higher odds of quitting tobacco at 3 months (OR=2.13; 95% CI: 1.02–4.46). Dual and poly users aged <21 years at baseline who received the BTI+AG intervention had higher odds of reducing the number of tobacco products used at 3 months (OR=2.94; 95% CI: 1.03–8.37).

**CONCLUSIONS:**

The BTI was effective for people aged 18–20 years. The current study offers insight into components of interventions that might be successful in helping this age group decrease tobacco use.

## INTRODUCTION

Tobacco use is increasing among young people in the US, with 12.5% of middle school and 27.5% of high school students reporting past 30-day use of a tobacco product in 2019^1^. This is an increase of approximately 1.32 million youth from the previous year, largely driven by the rise in electronic nicotine delivery systems (ENDS)^2^. Young adults (aged 18–24 years) also report more than twice the national average of current ENDS use compared to adults in the US (7.6% vs 3.2%, respectively)^3^. Overall, 17.1% of those aged 18–24 years report currently using a tobacco product, with 11.2% using a combustible product^3^. Further, the use of multiple tobacco products is more prevalent among young adults than mono use^4^. Unfortunately, young people who use multiple products report higher nicotine dependence, greater difficulty quitting, and a greater likelihood of transitioning to other tobacco products^5-7^.

Tobacco addiction is established early in life. Individuals who begin regular smoking between the ages of 18 to 20 years have lower odds of both intending and attempting to quit as well as higher odds of nicotine dependence compared to individuals who begin smoking after the age of 21 years^8^. Indeed, 90% of smokers begin smoking prior to the age of 18 years and 99% begin smoking before the age of 25 years^5^. Further, only half of daily adult smokers report daily smoking before the age of 18 years, but the majority (85%) report daily smoking by the age of 21 years^9^. These data led lawmakers to pursue legislation increasing the minimum age to purchase tobacco to 21 years of age^10^. This is particularly salient for military trainees, most of whom are under 21 years of age and also have historically higher rates of tobacco use compared to the general population^11-13^.

Within the US Air Force, approximately 5.9% of Airmen trainees (called such regardless of sex or gender identity) report current use of cigarettes, 2.1% current use of smokeless tobacco, 2.2% current use of hookah, and 15.3% current use of ENDS^13^. These rates are likely driven by a long history of targeted tobacco advertising and promotion on military bases^14,15^, a culture that supports tobacco use^16^, and the availability of cheap tobacco products on the base^17^. Previous studies have documented that the training year is a particularly high-risk period for tobacco use, with 7.9% to 12.4% of never users initiating tobacco products for the first time during this time^18,19^. Furthermore, the average age of trainees who report current tobacco use is 20 years^13^. Thus, the majority of these young adult tobacco users would be impacted by increased age restrictions on tobacco use.

On 20 December 2019, the federal minimum age for the sale of tobacco products was raised from 18 years to 21 years^9^. This legislation, known as ‘Tobacco 21’, instantly made it illegal for retailers in states without existing Tobacco 21 laws to sell tobacco to anyone under 21 years^9^. Evidence from local jurisdictions has demonstrated that Tobacco 21 is an effective tobacco control strategy^20,21^, supported by the majority of US adults^22^. However, this legislation does nothing to help the existing large number of people aged 18–20 years with established nicotine dependence to quit, particularly given that they were previously allowed to legally purchase tobacco prior to the passages of Tobacco 21^23^.

Even though many young people who initiate tobacco use want to quit within a short time of commencing^24^, evidence suggests that nicotine addiction occurs very quickly in this population, making the odds of a successful unaided quit attempt very difficult^25^. While cessation at a younger age is associated with better health outcomes and less mortality^26^, there is limited evidence for the long-term effectiveness of tobacco cessation programs for young adults^27,28^. In a Cochrane review of smoking cessation interventions for young adults, Fanshawe et al.^28^ found limited evidence that behavioral and pharmacological smoking cessation interventions produced long-term smoking cessation. Interventions included in the review were varied, including individual or group counseling with and without self-help materials, pharmacological interventions, computer interventions, or messaging interventions^28^; however, group-based behavioral interventions were identified as the most promising intervention for young adults. Nevertheless, the authors concluded that the overall evidence on the effectiveness for smoking cessation interventions for young adults remains limited, suggesting a need for additional randomized controlled trials of smoking cessation interventions for this population^28^. Another limitation to existing smoking cessation interventions for young adults is that the majority of these programs focus on cigarettes, despite the growing popularity of non-cigarette tobacco products among young adults^27^. Therefore, it is critical that effective tobacco cessation programs are developed addressing the wide range of currently available tobacco products and contemporary patterns of use. Further, these reviews^27,28^ included studies among young adults (aged 18–24 years); thus, less information is known about effective interventions specifically among young adults between 18 and 20 years.

The current study sought to fill this gap by testing the efficacy of a group-based Brief Tobacco Intervention (BTI) as a tobacco cessation program among a diverse sample of non-college attending young adults who recently enlisted in the US Air Force. We specifically examined whether the BTI, which previously produced null results when tested with a sample of young adults^29^, was effective for people aged 18–20 years specifically, who we know from the literature are less likely to quit^8^, and, importantly, are now legally unable to purchase tobacco^10^ making the need to quit even more salient in this population.

## METHODS

### Study design

A description of the clinical trial and interventions can be found elsewhere^29,30^. Briefly, this study was a three-group clustered randomized clinical trial. Participants were randomized by squadron (groups of about 50 Airmen who undergo all training and education together) to one of three conditions: 1) BTI + Airman’s Guide to Remaining Tobacco Free (AG), a relapse prevention pamphlet, 2) AG intervention alone, or 3) the National Cancer Institute’s Clearing the Air (CTA) pamphlet, a standard smoking cessation intervention. The outcome for the primary and secondary analyses was the use of tobacco products at follow-up at 3 months.

### Participants

Participants were US Air Force Airmen undergoing Technical Training at Joint Base San Antonio-Lackland Air Force Base in San Antonio, Texas, from April 2017 through January 2018. Among the 3347 participants that were approached, 2999 consented to participate (89.6% consent rate). Eligibility criteria included being at least 18 years of age and understanding the consent process in English. Among those, 2969 were eligible to participate in the study and 2117 (71.3%) were aged 18–20 years. We completed the follow-up at 3 months with 2611 Airmen (87.9% follow-up rate). The protocol was approved by the Institutional Review Board at the 59th Medical Wing of the US Air Force.

### Procedure

Airmen were convened by squadrons in groups of approximately 50 Airmen per intervention. Upon arrival, the study and procedures were described, and Airmen were given an opportunity to ask questions. After obtaining informed consent, Airmen were administered a pre-test assessment. All Airmen received one of the interventions (BTI+ AG, AG, or CTA), regardless of consent status since these interventions were considered part of Air Force training. Airmen assigned to receive the AG or CTA were provided with a 5-minute discussion of the key concepts in the booklets. Airmen were encouraged to keep the booklets for the duration of Technical Training to use as a reference for themselves or a fellow Airmen. Those who were randomized to the treatment condition (i.e. BTI + AG) then received the BTI intervention components, which included a series of open-ended questions based on the principles of motivational interviewing^29,30^. The BTI addressed the most commonly used tobacco products by Airmen (e.g. cigarettes, smokeless tobacco, e-cigarettes, hookah, cigars, little cigars, and cigarillos). All intervention discussions were meant to be interactive, utilizing the Socratic teaching style and eliciting participation through the principles of motivational interviewing. Interventions were delivered by trained research staff (most with prior military experience) and lasted approximately 45 minutes. After the delivery of the interventions, all consented Airmen completed the post-test assessment. During the last week of Technical Training (3 months after receiving the intervention), consented Airmen were reconvened by team to complete the follow-up assessment at 3 months, in groups of approximately 50 Airmen.

### Study measures

Tobacco use was assessed at baseline and at follow-up. Participants were asked how often they used the following products: cigarettes/roll-your-own cigarettes, smokeless tobacco/snus, cigars, cigarillos/ little cigars, pipe, ENDS, and hookah. Response categories ranged from: ‘Never’, ‘Quit’, ‘Less than monthly’, ‘Monthly’, ‘Weekly’, to ‘Daily’. Due to the fact that all Airmen are required to be tobacco free during Basic Military Training, at baseline, the questionnaire assessed tobacco use prior to Basic Military Training. For the primary outcome, tobacco use included the use of any tobacco product at the follow-up at 3 months. Tobacco product use at baseline and the follow-up at 3 months was defined as: regular mono use of any product, regular dual or poly use of any products (use of two or more products), seldom use of any product(s), and non-use of any products. Regular use refers to at least monthly use and the seldom use refers to less than monthly^3^.

### Statistical analyses

#### Primary analysis

To assess the efficacy of the BTI+AG or AG compared to CTA in preventing tobacco use, a multinomial logistic regression model was used to test both the intervention arms and baseline tobacco use status main effects as well as interaction effects between the intervention arms and baseline tobacco use status. The model adjusted for participant demographics (e.g. gender, race, education level, and marital status), as well as correlations between Airmen from the same squadron due to the group-based (cluster samples) randomization using Taylor series variance estimation method. Because we were primarily interested in the subsample of Airmen who were aged <21 years, a domain analysis of the multinomial logistic regression model was employed to incorporate the variability of the formation of different domains of age groups into the variance estimation (SAS Proc Surveylogistic). The overall ability of the multinomial logistic regression model to discriminate between the four tobacco use categories was quantified by estimating nonparametric polytomous discrimination index, bootstrapped 95% confidence intervals^31,32^, and pairwise C-statistics^33^ between categories to determine which categories can be well discriminated. The significance level was specified at alpha=0.05. All analyses were performed in SASv9.4 (Cary, NC, USA) and R3.6.0 (The R Foundation for Statistical Computing).

#### Secondary analysis

Since ENDS use among young adults has increased dramatically in recent years^3^, we conducted a secondary analysis to determine whether the intervention produced cessation or harm reduction effects among ENDS users specifically at baseline (i.e. regular mono users of ENDS, and dual or poly users of ENDS and other products). A similar analytical approach as described for the primary analysis was used.

## RESULTS

The majority of participants were White males, and roughly 20% of participants were Hispanic (Table 1). Regular mono ENDS use at baseline and regular mono tobacco and ENDS use at follow-up were higher among participants aged <21 years compared to those aged >21 years.

**Table 1:** Participants’ characteristics by interventions and age groups

*Characteristics*	*Age <21 years (n=2117)*			*Age ≥21 years (n=852)*		
	*BTI+AG (n=1034) n (%)*	*AG (n=566) n (%)*	*CTA (n=517) n (%)*	*BTI+AG (n=404) n (%)*	*AG (n=205) n (%)*	*CTA (n=243) n (%)*
Age (years)*	18.8 (18;19;19)	18.8 (18;19;19)	18.8 (18;19;19)	23.4 (21;22;24)	23.6 (21;23;24)	23.4 (21;22;25)
**Male**	728 (70.4)	369 (65.2)	385 (74.5)	271 (67.1)	149 (72.7)	173 (71.2)
**Race**						
Black	202 (19.5)	110 (19.4)	94 (18.2)	87 (21.5)	30 (14.6)	58 (23.9)
White	652 (63.1)	348 (61.5)	339 (65.6)	237 (58.7)	126 (61.5)	133 (54.7)
Multi-race	106 (10.3)	64 (11.3)	53 (10.3)	40 (9.9)	22 (10.7)	24 (9.9)
Other	74 (7.2)	44 (7.8)	31 (6.0)	40 (9.9)	27 (13.2)	28 (11.5)
Hispanic	220 (21.3)	106 (18.7)	103 (19.9)	94 (23.3)	50 (24.4)	57 (23.5)
**Married**	49 (4.7)	23 (4.1)	21 (4.1)	92 (22.8)	41 (20.0)	45 (18.5)
**Education level**						
High school diploma/GED	795 (76.9)	418 (73.9)	407 (78.7)	107 (26.5)	71 (34.6)	75 (30.9)
Vocational training	11 (1.1)	1 (0.2)	1 (0.2)	0 (0.0)	6 (2.9)	3 (1.25)
Some college/associate’s	226 (21.9)	134 (23.7)	105 (20.3)	219 (54.2)	101 (49.3)	121 (49.8)
Bachelor’s degree or higher	2 (0.2)	13 (2.3)	4 (0.8)	78 (19.3)	27 (13.2)	44 (18.1)
**Military rank**						
Active duty	940 (91.3)	503 (89.8)	456 (88.4)	322 (80.1)	167 (82.7)	189 (78.8)
Guard	67 (6.5)	43 (7.7)	44 (9.5)	54 (13.4)	23 (11.4)	28 (11.7)
Reserve	23 (2.2)	14 (2.5)	11 (2.1)	26 (20.5)	12 (5.9)	23 (9.6)
**Prior tobacco use**						
Regular mono use	136 (13.2)	69 (12.2)	60 (11.6)	39 (9.7)	29 (14.2)	32 (13.2)
Regular dual/poly use	121 (11.7)	69 (12.2)	73 (14.2)	41 (10.2)	15 (7.4)	24 (9.9)
Seldom use	102 (9.9)	59 (10.4)	43 (8.3)	50 (12.4)	17 (8.3)	24 (9.9)
Non-use	673 (65.2)	368 (65.1)	340 (65.9)	274 (67.8)	143 (70.1)	163 (67.1)
**Prior ENDS use**						
Regular ENDS mono use	69 (7.0)	36 (6.4)	27 (5.2)	10 (2.5)	8 (3.9)	8 (3.3)
Regular concurrent use of ENDS and other products	75 (7.3)	50 (8.9)	56 (10.9)	23 (5.7)	7 (3.4)	15 (6.2)
Regular other products use	113 (11.0)	52 (9.2)	50 (9.7)	47 (11.6)	29 (14.2)	33 (13.6)
Seldom use	102 (9.9)	59 (10.4)	43 (8.3)	50 (12.4)	17 (8.3)	24 (9.9)
Non-use	673 (65.2)	368 (65.1)	340 (65.9)	274 (67.8)	143 (70.1)	163 (67.1)
**Tobacco use at 3 months**						
Regular mono use	80 (8.6)	43 (8.9)	41 (9.0)	28 (7.9)	9 (5.1)	12 (5.6)
Regular dual/poly use	78 (8.4)	34 (7.0)	43 (9.5)	16 (4.5)	7 (4.0)	12 (5.6)
Seldom use	58 (6.3)	30 (6.2)	23 (5.1)	27 (7.6)	12 (6.9)	15 (7.0)
Non-use	711 (76.7)	377 (77.9)	348 (76.5)	285 (80.1)	147 (84.0)	175 (81.8)
**ENDS use at 3 months**						
Regular ENDS mono use	49 (5.3)	26 (5.4)	21 (4.6)	11 (3.1)	4 (2.3)	3 (1.4)
Regular concurrent use of ENDS and other products	59 (6.4)	24 (5.0)	27 (5.9)	7 (2.0)	5 (2.3)	9 (4.2)
Regular other products use	50 (5.4)	27 (5.6)	36 (7.9)	26 (7.3)	7 (4.0)	12 (5.6)
Seldom use	58 (6.3)	30 (6.2)	23 (5.1)	27 (7.6)	12 (6.9)	15 (7.0)
Non-use	711 (76.7)	377 (77.9)	348 (76.5)	285 (80.1)	147 (84.0)	175 (81.8)

* Mean (1st quartile; median; 3rd quartile). BTI: brief tobacco intervention. CTA: national cancer institute’s clearing the air intervention. AG: airman’s guide intervention. GED: general educational development.

### Primary analysis

From the domain analysis of the multivariable multinomial logistic regression model, there were significant interaction effects between the interventions and baseline tobacco use (Wald χ^2^=150.4, df=18, p<0.0001) and baseline tobacco use main effects (Wald χ^2^=394.2, df=9, p<0.0001), although the main effect of the interventions was not significant (Wald χ^2^=3.1, df=6, p=0.794). Compared to CTA, BTI+AG intervention demonstrated significant efficacy in helping younger Airmen (aged <21 years) who were regular mono users at baseline to quit at the follow-up at 3 months (OR=2.13; 95% CI: 1.02–4.46, p=0.045) (Table 2). Regular dual/ poly users at baseline who received the BTI+AG compared to CTA were less likely to quit at follow-up compared to mono users (OR=0.36; 95% CI: 0.15–0.91, p=0.030). Comparing the BTI+AG with CTA, regular dual/poly users at baseline were less likely to report regular dual/poly use at the follow-up at 3 months, compared to mono users (OR=0.34; 95% CI: 0.12–0.97, p=0.044) (Table 2). In other words, the BTI+AG helped dual/poly users at baseline to become mono users at follow-up. The AG alone did not show any significant intervention effects compared to CTA (p>0.05). There were no significant differences between the intervention groups in the group of people aged >21 years (Supplementary file Table 1).

**Table 2:** Primary analysis predicting intervention effects on use of tobacco products at follow-up, among participants aged <21 years

*Tobacco product use at baseline*	*Intervention arm*	*Tobacco product use at 3 months OR (95% CI)*	
		*Non-use*	*Regular dual or poly use*
Any regular tobacco mono use (Ref.)	BTI+AG vs CTA	**2.13 (1.02–4.46)**	1.63 (0.54–4.89)
	AG vs CTA	2.07 (0.81–5.26)	1.54 (0.54–4.40)
Regular dual or poly tobacco use	BTI+AG vs CTA	**0.36 (0.15–0.91)**	**0.34 (0.12–0.97)**
	AG vs CTA	0.46 (0.15–1.40)	0.37 (0.12–1.09)

Bold indicates statistical significance at p<0.05.

### Secondary analysis

There were significant interaction effects between the interventions and baseline ENDS use (Wald χ^2^=201.4, df=24, p<0.0001) and baseline ENDS use main effects (Wald χ^2^=457.4, df=12, p<0.0001), although again the main effect of the intervention was not significant (Wald χ^2^=2.7, df=6, p=0.851) from the domain (Airmen who were aged 18–20 years) analysis of the multivariable multinomial logistic regression model. Among regular ENDS mono users at baseline, younger Airmen (aged <21 years) in the BTI+AG intervention were more likely to report abstinence at the follow-up compared to Airmen receiving CTA (OR=2.95; 95% CI: 1.16–7.53, p=0.024) (Table 3). Regular concurrent ENDS and other tobacco product users who received the BTI+AG compared to CTA at baseline were less likely to quit at follow-up compared to reporting mono use (OR=0.19; 95% CI: 0.05–0.69, p=0.011), in other words, they were more likely to become mono users at follow-up. The same was true for participants who received the AG (OR=0.18; 95% CI: 0.04–0.79, p=0.023). There were no significant differences between the intervention groups in the group of people aged >21 years (Supplementary file Table 2).

**Table 3:** Secondary analysis predicting intervention effects on use of tobacco products at follow-up among ENDS users at baseline, among participants aged <21 years

*Any ENDS use at baseline*	*Intervention arm*	*Tobacco product use at 3 months OR (95% CI)*	
		*Non-use*	*Regular dual or poly use*
Regular mono use of ENDS (Ref.)	BTI+AG vs CTA	**2.95 (1.16–7.53)**	1.67 (0.37–7.53)
	AG vs CTA	2.53 (0.81–7.91)	1.27 (0.25–6.55)
Regular concurrent use of ENDS and other products	BTI+AG vs CTA	**0.19 (0.05–0.69)**	0.28 (0.08–1.00)
	AG vs CTA	**0.18 (0.04–0.79)**	0.26 (0.07–1.03)

Bold indicates statistical significance at p<0.05.

The primary outcome has four categories (regular mono use of a product, regular dual or poly use of any products, seldom use of any product(s), and non-use of any products). Thus, the null polytomous discrimination index (PDI) of the overall model was 0.25 (i.e. a random guess). The estimated PDI of 0.43 (bootstrapped 95% confidence interval: 0.40–0.45) from the primary analysis model was about 1.7 times of the lower bound, which corresponds to no discriminative ability, indicating the model has fairly good predictive discriminative ability. The pairwise C-statistic of 0.76 for the comparison of ‘regular mono use of any product’ and ‘non-use’ categories at follow-up at 3 months, and a value of 0.84 for the comparison of ‘regular dual or poly of any products’ and ‘non-use’ categories indicated that the model has good to excellent discriminative ability for the comparisons of the primary interests.

## DISCUSSION

This study found that a brief 45-minute tobacco intervention was effective in reducing tobacco use among a large military sample of racially diverse young adults. Specifically, the BTI + AG intervention demonstrated significant efficacy in helping younger participants (aged <21 years) who were mono tobacco users at baseline (i.e. cigarettes/roll-your-own cigarettes, smokeless tobacco/snus, cigars, cigarillos/little cigars, pipe, ENDS, or hookah) to quit at the follow-up at 3 months, as well as dual and poly tobacco users reduce the number of tobacco products they used and transition to mono use. Additionally, among exclusively mono ENDS users at baseline, these individuals were more likely to have quit ENDS at follow-up if they received the BTI + AG intervention, while dual and poly users were more likely to reduce to mono use. These results are promising because despite ENDS use being on the rise among young adults^1-3^, there has been limited evidence for the long-term effectiveness of ENDS cessation programs for this age group^27,28^. While recent Tobacco 21 legislation should curb the uptake of tobacco use among individuals aged <21 years, this policy does not address cessation efforts among youth who have already developed a nicotine dependence from these products^23^. Therefore, the efficacy of this BTI + AG intervention has implications for cessation efforts for youth and young adults using tobacco, including those exclusively using ENDS.

Although ENDS are the most common tobacco product used among young adults^3^, including individuals in Air Force training^13^, few youth-focused cessation programs have focused specifically on helping ENDS users quit these products^34^. In the current study, we observed reductions in tobacco use among mono and dual/poly ENDS users who received the BTI + AG intervention. Given that ENDS deliver nicotine at higher or comparable levels to cigarettes, are capable of introducing nicotine dependence to otherwise tobacco naïve individuals, and have negative cardiovascular health effects (e.g. elevating heart rate and diastolic blood pressure)^35-37^, identifying interventions that effectively reduce ENDS use is important to spur future ENDS cessation endeavors.

There are multiple components of the current BTI + AG intervention that likely facilitated reducing tobacco use in this population. For example, the BTI intervention focused on restructuring cognitive misperceptions related to tobacco use (e.g. normative beliefs and perceptions of harm) and decreasing hyperbolic discounting (i.e. consider long-term goals in the context of current behavior)^38^. Clear communication addressing misperceptions about tobacco harms might have been particularly useful for those using ENDS, given that young people have reported fatigue and confusion in regard to conflicting information about the risk of ENDS^39^. Further, having these young adults identify the ways in which tobacco use might impact their long-term goals might have helped facilitate quitting similarly to other substance use interventions^38^. Additionally, the BTI intervention was interactive and group-based, with approximately 50 participants per group. A Cochrane review among young adults found that group-based tobacco interventions had the most promising results for reducing tobacco use rates^28^. Thus, current findings are consistent with the civilian literature in suggesting that youth are more likely to quit when hearing supportive responses and feedback from their peers during the intervention^28,34^. Further, the interventionists in this study had a military career background. Thus, it is likely that an intervention facilitated by someone trusted and respected by these young adults was more impactful to them. Although there were likely multiple components of this intervention that helped reduce tobacco use, it is unclear which specific components were more effective than others. It will be important for future studies to continue examining specific strategies that help individuals aged <21 years quit tobacco products.

It remains unclear as to why the intervention was effective for individuals aged <21 years but not ≥21 years. It is possible that those aged <21 years have been using tobacco products for a shorter time compared to their older counterparts, and thus have a lower level of nicotine dependence. As mentioned previously, only half of daily smokers are smoking daily before the age of 18 years, but 85% are smoking daily by the age of 21 years^9^. However, there is evidence to suggest that nicotine dependence can occur within days to weeks of the onset of occasional use among adolescents^40^. More research is needed to better understand why these differences in treatment effects may exist. Additionally, future studies should consider controlling for nicotine dependence in the evaluation of youth and young adult tobacco cessation programs.

The BTI + AG was effective in promoting mono use at the follow-up at 3 months among younger participants (<21 years) who were dual and poly tobacco users at baseline. While ultimately the goal of any tobacco cessation intervention is total abstinence, given that dual and poly use is more prevalent among young adults than mono use^4^, an intervention such as the BTI + AG, could still be beneficial for poly tobacco users to support their transition to mono use. The Theory of Planned Behavior hypothesizes that attitudes toward behavior, subjective norms, and perceived behavioral control shape an individual’s intentions and behaviors^41^. Therefore, one would expect that if perceived behavioral control over one’s tobacco use is established, individuals could build on this initial success to work towards complete tobacco abstinence long-term. Given the fact that poly tobacco users exhibit higher levels of nicotine dependence compared to mono users^42^, any effects on tobacco use reduction from a brief intervention is promising. Future interventions could consider building upon the BTI + AG with additional treatment components (e.g. automated text messaging, booster session) to strengthen the cessation effect for poly tobacco users, as there is evidence to suggest that adding text-messaging to other tobacco cessation interventions can increase quit rates by 50–60%^43^.

### Limitations

There are some limitations that are important to consider. This cessation intervention was delivered during an enforced military tobacco ban, which has been found to produce long-term cessation rates from 15 to 20% in military populations^44-46^. However, all Airmen across all three randomized arms would have experienced the same ban, and therefore the ban would not be expected to confound results. Further, even though an 8½-week enforced tobacco ban is unique to the military, tobacco bans are not unlike smoke-free policies and tobacco restrictions on college campuses and dorms, which have also been shown to reduce tobacco prevalence rates^47^. Because baseline tobacco rates relied on self-report of tobacco use prior to the tobacco ban, there may be some social desirability bias, but this was unlikely to differ by intervention arm and therefore would not be anticipated to have a differential effect on our outcomes of interest. Additionally, self-reported tobacco abstinence at follow-up was not biochemically validated. However, this bias should have affected all three treatment conditions equally given that there was not a no-treatment control arm.

The current study was not powered to detect racial and ethnic differences in the efficacy of the BTI + AG intervention. However, race was one of the covariates included in our primary and secondary analysis models, and the direct effect of race on our primary outcome was non-significant (p=0.107 for the primary analysis and p=0.072 for the secondary analysis, respectively). Given racial and ethnic disparities in tobacco use, future studies should consider powering clinical trials to examine potential racial and ethnic differences in program efficacy.

Finally, this population included Air Force personnel and thus results might not be generalizable to other military branches despite similar tobacco bans across all military training. It may also not be completely generalizable to civilian populations. However, this intervention could easily be adapted to a community technical school setting, where large numbers of non-college attending young adults receive career and vocational training, similar to the study population. Additional opportunities for translation of the BTI + AG include adapting the intervention for high school youth in order to prevent transitions to nicotine dependence early on for those tobacco users.

## CONCLUSIONS

The current study identified the efficacy of a group-based brief tobacco intervention in reducing tobacco rates at the follow-up at 3 months among a sample of young adults (aged 18–20 years) recently enlisted in the US Air Force. Although the intervention was implemented within a military sample, there are important implications for both civilian and military young adults. First, this population offered a unique opportunity to examine a cessation intervention within a large, racially diverse, non-college sample of young adults. Secondly, despite rising tobacco rates in young adulthood^1-3^, there have been few randomized controlled trials of tobacco cessation interventions in this age group^27,28^. Finally, cessation trials have typically focused on cigarettes and fewer have observed outcomes among young adults using only ENDS. Therefore, the current cessation trial expands upon the prevention efforts of Tobacco 21 laws, by offering effective strategies for young adults (aged 18–20 years) who are already established tobacco users (including ENDS only users), to quit their use of these products.

## Data Availability

The data supporting this research are available from the authors on reasonable request.
