# Supplementary material for: Efficacy of a group-based brief tobacco intervention among young adults aged 18–20 years in the US Air Force
Source: Tob Induc Dis. 2021 Dec 8;19:95. doi: 10.18332/tid/143282 (PMC8653010; doi:10.18332/tid/143282)
Supplement: Supplementary file 1 [file TID-19-95-s1.pdf]

Supplementary Table 1. Predicting intervention effects on use of tobacco products at follow-up (among participants ≥21 years of age)

Tobacco Product Use at Baseline	Intervention arm	Tobacco Product Use at 3-month (reference category: Regular Mono Use) OR (95% CI)	
		No use	Regular dual or poly use
Any regular tobacco mono use	BTI+AG vs. CTA	1.48 (0.32, 6.74)	0.66 (0.06, 7.44)
	AG vs. CTA	3.20 (0.51, 19.97)	2.74 (0.38, 19.50)
Regular dual or poly tobacco use	BTI+AG vs. CAT	0.88 (0.22, 3.56)	0.37 (0.11, 1.21)
	AG vs. CTA	1.12 (0.20, 6.38)	0.26 (0.05, 1.30)

Supplementary Table 2: Predicting intervention effects on use of tobacco products at follow-up among ENDS users at baseline (among participants ≥ 21 years of age)

Any ENDS Use at Baseline	Intervention arm	Tobacco Product Use at 3-month (reference category: Regular Mono Use) OR (95% CI)	
		No use	Regular dual or poly use
Regular mono use of ENDS	BTI+AG vs. CTA	0.51 (0.03, 8.22)	0.25 (0.01, 8.00)
	AG vs. CTA	NA	NA
Regular concurrent use of ENDS and other products	BTI+AG vs. CTA	1.02 (0.20, 5.29)	0.42 (0.06, 3.11)
	AG vs. CTA	0.87 (0.09, 8.01)	NA

NA: not sufficient information for estimation.

© 2021 Little M.A. et al.
